# PANCREATODUODENECTOMY IN PATIENT WITH VON HIPPEL-LINDAU DISEASE: A LITERATURE REVIEW

**DOI:** 10.1590/0102-672020220002e1697

**Published:** 2022-11-25

**Authors:** José Marcus Raso Eulálio, Thales Penna Carvalho, Eloá Pereira Brabo, Antonio Luis Eiras Araújo, Adriana de Oliveira Eulálio, Felipe Nogueira Beirão, José Eduardo Ferreira Manso

**Affiliations:** 1 Universidade Federal do Rio de Janeiro, Department of Surgery – Rio de Janeiro (RJ), Brazil; 2 University Hospital Clementino Fraga Filho, Oncology Service – Rio de Janeiro (RJ), Brazil; 3 University Hospital Clementino Fraga Filho, Radiodiagnosis Service – Rio de Janeiro (RJ), Brazil; 4 National Institute of Traumatology and Orthopedics, Anestesiology Service – Rio de Janeiro (RJ), Brazil; 5 University Hospital Clementino Fraga Filho, Anestesiology Service – Rio de Janeiro (RJ), Brazil.

**Keywords:** Pancreaticoduodenectomy, von Hippel-Lindau Disease, Neuroendocrine Tumors, Pancreaticoduodenectomia, Neoplasias Pancreáticas, Doença de von Hippel-Lindau

## Abstract

**BACKGROUND:**

The von Hippel-Lindau disease is a highly penetrant autosomal dominant syndrome characterized by tumor predisposition in different organs.

**AIM:**

This study aimed to describe a case of a pancreatoduodenectomy for a 30-year-old male patient with von Hippel-Lindau disease.

**METHODS:**

We present a case study and the literature review aiming at the state-of-the-art management of a patient with pheochromocytoma, capillary hemangioblastoma in the peripheral retina, and two neuroendocrine tumors in the pancreas.

**RESULTS:**

A larger pancreatic lesion was located in the uncinate process, measuring 31 mm. The smaller lesion was located in the proximal pancreas and was detected only on the positron emission tomography-computed tomography scan with DOTATOC-68Ga. Genetic investigation revealed a mutation in the locus NM_000551.3 c.482G>A (p.Arg161Gln) of the Von Hippel-Lindau Human Suppressor gene. The uncinate process tumor was larger than 30 mm and the patient had a mutation on exon 3; therefore, we indicated a pancreatoduodenectomy involving the proximal pancreas to resect both tumors *en bloc*. During the postoperative period, the patient presented a peripancreatic fluid collection, which was treated as a grade B pancreatic fistula with clinical resolution of the complication. On postoperative day 21, he was discharged home.

**CONCLUSION:**

The management of patients with von Hippel-Lindau disease and pancreatic neuroendocrine tumors is complex and must be centered on tertiary institutions with a large volume of pancreatic surgery. Although the current literature assists in decision-making in most situations, each step of the treatment requires analysis and discussion between different medical specialties, including surgeons, clinicians, radiologists, and anesthesiologists.

## RESUMO

**RACIONAL:**

A doença de von Hippel Lindau é uma síndrome autossômica dominante que se caracteriza por maior incidência de tumores em diferentes órgãos.

**OBJETIVO:**

Descrever um caso de pancreatoduodenectomia em paciente do sexo masculino de 30 anos com von Hippel Lindau.

**MÉTODO:**

Apresentamos o caso e a revisão da literatura realizada para otimizar o manejo do paciente, que apresentava feocromocitoma, hemangioblastoma capilar na retina periférica e dois tumores neuroendócrinos no pâncreas.

**RESULTADOS:**

O maior tumor pancreático localizava-se no processo uncinado medindo 31 mm. A lesão menor estava localizada no corpo proximal do pâncreas e foi detectada apenas na tomografia computadorizada por emissão de pósitrons com DOTATOC-68Ga. A investigação genética revelou uma mutação no locus NM_000551.3 c.482G>A (p.Arg161Gln) do gene supressor humano de Von Hippel-Lindau. O tumor no processo era maior que 30mm e o paciente apresentava mutação no exon 3. Indicamos pancreatoduodenectomia envolvendo o corpo proximal do pâncreas para ressecar em bloco ambos os tumores. No pós-operatório o paciente apresentou coleção líquida peripancreática que foi tratada como fístula pancreática grau B, com resolução clínica da complicação. Ele recebeu alta hospitalar no vigésimo primeiro dia pós-operatório.

**CONCLUSÕES:**

o manejo de pacientes com doença de von Hippel Lindau e tumores neuroendócrinos pancreáticos é complexo e deve ser centrado em instituições terciárias com grande volume de cirurgia pancreática. Embora a literatura atual auxilie na tomada de decisão na maioria das situações, cada etapa do tratamento requer análise e discussão entre diferentes especialidades médicas, incluindo cirurgiões, clínicos, radiologistas e anestesiologistas.

## INTRODUCTION

The von Hippel-Lindau (VHL) disease is a highly penetrant autosomal dominant syndrome characterized by tumor predisposition in different organs, such as the central nervous system, kidneys, pancreas, and adrenals. Clinically, it is classified as type I (without pheochromocytoma) and II (with pheochromocytoma). Group II is subclassified into A (with kidney tumor), B (without kidney tumor but with tumors in other organs), and C (without other tumors)^26^.

The VHL disease is caused by mutations in the VHL suppressor gene on the short arm of chromosome 3 (3p25-26). To date, there are more than 500 reported mutations related to the progression of the disease^13^.

The VHL suppressor gene has three exons: exon 1, with nucleotides 1–340 (codons 1–113); exon 2, with nucleotides 341–463 (codons 114–154); and exon 3, with nucleotides 464–642 (codons 155–213). The patient from the case reported ahead has the mutation c.482G>A (p.Arg161Gln). In this case, the substitution of the nucleobase guanine for alanine on nucleotide 482 causes the switching of the amino acids arginine for glycine on codon 161 (exon 3). The resultant tumor suppressor protein has its function lost or altered^22^. Recently, Hong et al. described mutations in 80 Eastern families on the three exons and even in the intron connecting exons 2 and 3^13^.

The case presented in this review is a type IIB VHL disease, as he had a bilateral pheochromocytoma, two pancreatic neuroendocrine tumors (NETs), and capillary hemangioblastoma in the peripheral retina of the left eye.

### Case report

We present a case of a 30-year-old male patient who underwent right laparoscopic adrenalectomy and left laparoscopic adrenalectomy when he was 16 and 19 years old, respectively, due to pheochromocytoma.

During the follow-up at the endocrinology service, the patient reported vision loss in the left eye. Ophthalmological investigation revealed 80% vision loss in the affected eye and discovered a capillary hemangioblastoma in the peripheral retina, which is a benign vascular proliferative tumor associated with the VHL disease. He also complained of sporadic mild morning diarrhea. He did not refer to any respiratory symptoms. The patient was on 10 mg prednisone and 20 mg fluoxetine daily.

His medical and social history also includes one episode of infectious endocarditis at 14 years old and mumps at an unrecorded age. The patient also has a seven-pack-year tobacco smoking history. He has a high school degree, works as a clerk, and is married without children.

A family investigation revealed the patient's father had the same mutation. Further research revealed he had asymptomatic pheochromocytoma and pancreatic NETs.

Physical examination revealed normal vital signs. Normal breath sounds on auscultation and nothing unusual on the abdominal examination, except for the scars of the previous laparoscopic procedures. Height: 1.65 m; weight: 60.0 kg; body mass index: 22.0 kg/m^3^.

Genetic investigation revealed a mutation in the locus NM_000551.3 c.482G>A (p.Arg161Gln) of the Von Hippel-Lindau Human Suppressor gene, which confirms the diagnosis^22^. Other tumors and conditions associated with the VHL disease were then investigated.

Laboratory test findings were unremarkable. Serum aldosterone, insulin, gastrin, ACTH, renin, hemogram, and glucagon had normal values. Hepatic, pancreatic, and renal function markers were also normal.

**Figure 1:**
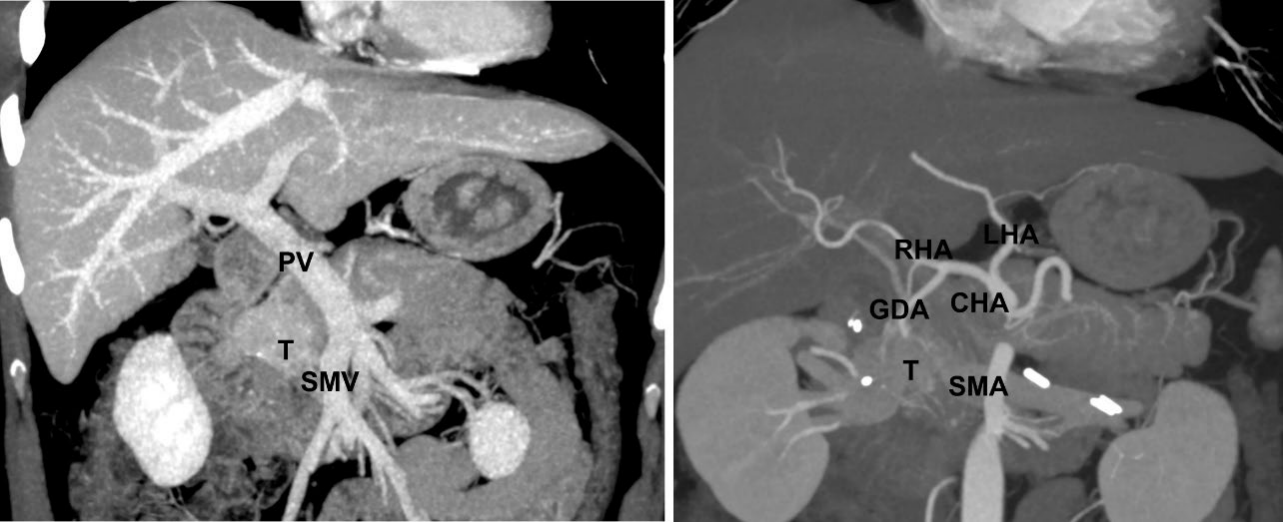
Angiotomography computed tomography scan. Venous and arterial reconstructions. The patient has an uncommon replaced gastroduodenal artery arising from the right hepatic artery, which not interfere with surgical planning. (T) tumor; (PV) portal vein; (SMV) superior mesenteric vein; (CHA) common hepatic artery; (SMA) superior mesenteric artery; (RHA) right hepatic artery; (LHA) left hepatic artery; (GDA) gastroduodenal artery.

Magnetic resonance imaging (MRI) showed a solid tumor measuring 31×19 mm in the uncinate process of the pancreas. Abdominal angiotomography using a 128-slice computed tomography (CT) scanner demonstrated the same lesion with a size of 31×23 mm, without any evidence of invasion of the mesenteric vessels or other adjacent structures (Figure 1).

Endoscopic ultrasound (EUS) described a solid lesion in the uncinate process, and the fine-needle biopsy revealed a grade 1 NET with Ki-67 <2%.

**Figure 2:**
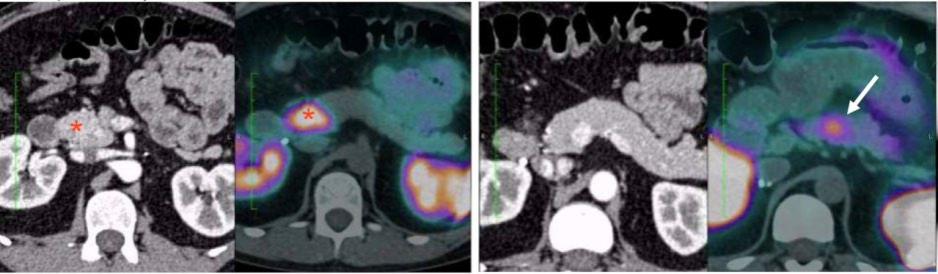
Positron emission tomography-computed tomography scan with DOTATOC-68Ga. Left: the uncinate process tumor in the computed tomography scan with the corresponding image on the Positron emission tomography-computed tomography (*). Right: the pancreatic body tumor is not viewable on the computed tomography scan but detected with the Positron emission tomography-computed tomography (arrow).

We completed the imaging evaluation with a positron emission tomography (PET) scan with DOTATOC-68Ga that revealed a hypervascular lesion in the head of the pancreas with a Maximum Standard Unit Value (SUV_max_) of 35.8 and another small hypervascular nodule in the body, with a SUV_max_ of 7.2 (Figure 2). It is noteworthy that the smaller nodule in the body of the pancreas was not viewable on MRI, CT, or EUS.

Three imaging modalities narrowly missed the actual size of the tumor, which is a foremost factor for surgical indication. MRI and CT referred a 31 mm lesion and the EUS referred a 28 mm lesion (Table 1). While the macroscopic pathological analysis measured 30 mm, the histopathological analysis measured its maximum diameter of 35 mm, greater than any imaging modality, MRI included.

**Table 1:** Measurement of the tumors in different tools. Positron emission tomography-computed tomography scan is not an adequate tool for evaluating tumor size.

Examinations	Uncinate process tumor measurement	Pancreatic body tumor measurement
MRI scan	31×19 mm	Not detected
EUS	28×17 mm	Not detected
PET-CT scan	SUV_max_=35.8	SUV_max_=7.2
CT scan	31×23 mm	Not detected
Macroscopic analysis	28×24 mm	5×4 mm
Microscopic analysis	35×25 mm	5×4 mm

MRI: magnetic resonance imaging; EUS: endoscopic ultrasound; PET: positron emission tomography; CT: computed tomography; SUV_max_: Maximum Standard Unit Value.

Pancreatoduodenectomy including part of the pancreas to remove the small nodule was indicated. The patient was a grade 2 in the American Society of Anesthesiologists (ASA) Physical Status Classification System. We concluded the clinical investigation for this patient in mid-July 2020, when our city and country were dealing with the first wave of the COVID-19 infection. The healthcare system in our city was overloaded and vaccines against COVID-19 were still unavailable. Therefore, we chose to defer the surgical procedure until our institution was back to its usual care routine and nosocomial COVID-19 infection was less likely. Singularly, the patient had a replaced gastroduodenal artery arising from the right hepatic artery, which did not interfere with the procedure (Figure 1).

We scheduled the procedure for early November 2020. However, in the week of the operation, his wife had a mild COVID-19 infection. Although the patient himself presented no upper respiratory tract symptoms and his RT-PCR COVID-19 test showed a negative result, the protocol of our institution at the time recommended postponing the surgical procedure for 21 days.

A midline laparotomy was performed under general anesthesia. A detailed assessment of the peritoneal cavity did not find any sign of metastatic disease. Two palpable masses were found in the uncinate process and pancreatic body, without invasion of neighboring structures and organs. Intraoperative ultrasonography evaluation confirmed those findings.

**Figure 3:**
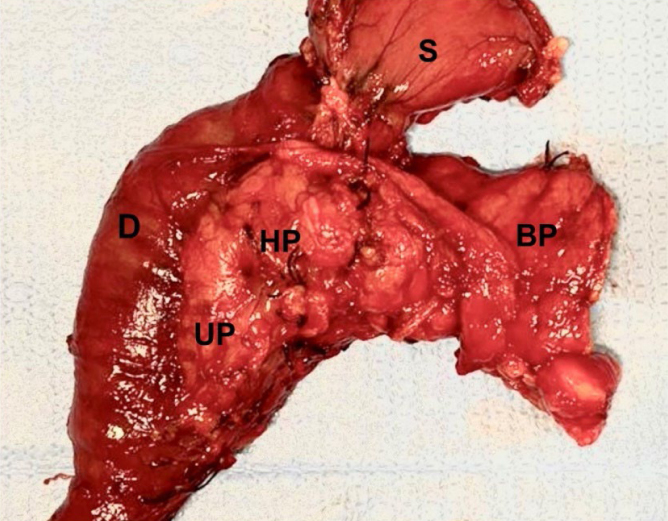
Surgical specimen. The tumors were palpable, but not viewable on the pancreatic surface. (S) distal stomach; (D) duodenum; (UP) uncinate process; (HP) head of the pancreas; (BP) proximal body of the pancreas.

We performed a pancreatoduodenectomy involving the distal stomach, duodenum, and the pancreas, from the uncinate process to the proximal body, with standard lymphadenectomy (Figure 3). For reconstruction, we created an invaginated pancreaticogastrostomy, an end-to-side hepaticojejunostomy, and end-to-side gastrojejunostomy with a single loop of proximal jejunum in a retrocolic manner. At the end of the procedure, we positioned two Blake 19Fr silicone drains in the peripancreatic and in the Morrison space. We also inserted a DobbHoff feeding tube through the nasal passage, placing its tip in the jejunum about 20 cm distal to the anastomoses.

The pancreatic remnant was mobilized distally to create an invaginated pancreaticogastrostomy. This is our pancreatic reconstruction technique of choice when we are operating on a soft parenchyma or a thin main duct, conditions the patient presented. Otherwise, we prefer duct-to-mucosa anastomosis, either with the stomach or with the jejunum. We also performed an end-to-side hepaticojejunostomy with separate stitches of polypropylene 5-0 aided by 2× magnification and an end-to-side gastrojejunostomy, using a single loop of bowel. We performed a standard lymphadenectomy, and the histopathological analysis retrieved 12 tumor-free lymph nodes.

Our patient did not have neurological symptoms or any signs of spinal lesions on imaging. The anesthesiology team indicated a lumbar epidural block, inserting a catheter for analgesia. They removed the catheter on the second postoperative day.

The postoperative period was uneventful until the second day, when the patient presented with a 38.0° axillar temperature associated with diminished breath sounds in the left chest. Other vital signs were normal. An x-ray and CT scan revealed opacification of the lower lobe of the left lung, suggesting atelectasis. We decided to lengthen the respiratory physical therapy exercises, which lowered his body temperature and improved his breathing pattern.

Drains were removed on postoperative days 2 and 3, as the drainage was below 10 ml in both. We routinely evaluate amylase in the drainage fluid after pancreatic resections and it was 30 IU in both drains, which was below the serum level.

**Figure 4:**
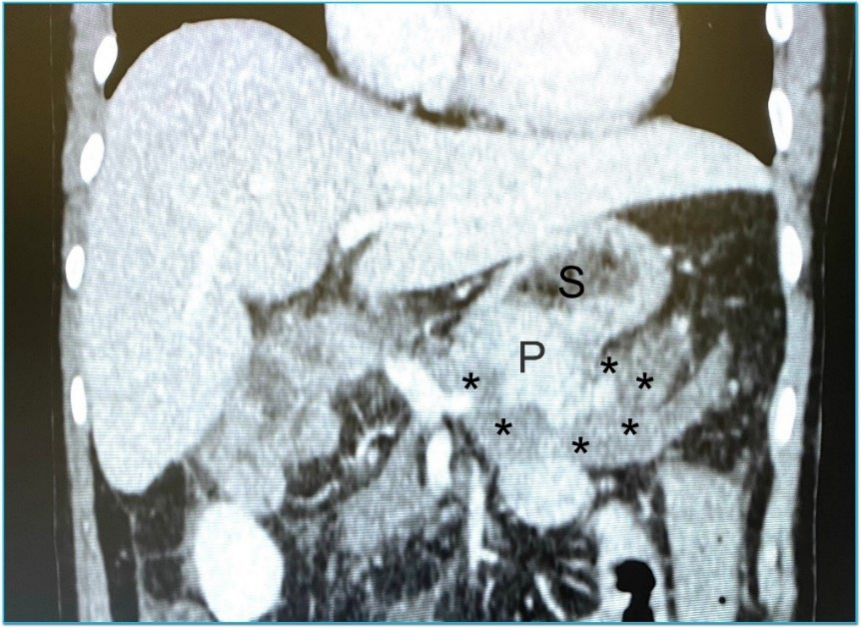
CT scan of a patient with nausea, vomiting, and leukocytosis on postoperative day 7. (S) stomach; (P) pancreas remnant. *Peripancreatic fluid.

Enteral diet through the feeding tube was started on day 2. On day 4, we began transitioning from enteral to oral feeding and released the patient from the intensive care unit for the surgical ward. On postoperative day 7, the patient presented nausea and emesis, associated with a 37.6° axillar temperature. The white cell count showed 18,600 leukocytes per milliliter of blood. Although there was no abdominal pain or distension, we decided for an abdomen CT scan, which revealed a small and undefined peripancreatic fluid collection (Figure 4). With the hypothesis of a pancreatic fistula, we withheld oral diet and resumed enteric feeding through the DobbHoff tube. We also added a large spectrum antibiotic (meropenem) and subcutaneous octreotide to the prescription.

A new CT scan for control performed on postoperative day 14 (day 7 of meropenem and octreotide use) revealed reduction of the peripancreatic fluid. Blood and urine cultures did not detect bacteria growth. The white blood cell count was normal. The patient was asymptomatic and nothing atypical was found on physical examination. We took out the DobbHoff tube and reintroduced oral feeding, which the patient tolerated very well. He was discharged home on postoperative day 21, after completing 14 days of meropenem, as recommended by the department of infectious diseases at our hospital. He went back to work 45 days after the operation. The patient is now asymptomatic and has maintained quarterly follow-ups with our surgical team for more than 1 year.

Apart from an atelectasis in the lower lobe of the left lung, the early postoperative period was uneventful until day 7. The discharge through the abdominal drains was below 10 ml in 24 h and with a low amylase concentration. All these conditions backed us in the decision of the early removal of the drains, as recommended by the Ottawa Pancreatic Drain Removal Algorithm^21^.

On day 7, the patient presented nausea, vomiting, hyperthermia, and leukocytosis. A CT scan revealed a small peripancreatic fluid accumulation. Although it was not a clearly defined abscess with rim enhancement on contrast, we decided to manage it as a pancreatic fistula with secondary infection. We instituted *nil per os*, enteral feeding through a DobbHoff tube, intravenous meropenem, and percutaneous somatostatin analog. As the collection was small and without clear limits, we chose not to drain it^1,15^. After 7 days, this fluid collection almost disappeared and there were no signs of persistent infection, so we reinitiated oral feeding.

We indicated broad-spectrum antibiotic with great pancreatic tissue penetration, considering the axillar temperature and the leukocytosis. Besides the recent pancreatoduodenectomy, the patient was glucocorticoids dependent, and we were concerned his clinical status could deteriorate fast with a possible ongoing severe intra-abdominal infection. Adachi et al., studying distal pancreatectomies, reported managing postoperative pancreatic fistulas with the association of carbapenem, octreotide, and protease inhibitor^1^.

Histopathological analysis confirmed two NETs in the uncinate process and body of the pancreas. Immunohistochemistry techniques asserted the diagnosis and quantified Ki-67 index <2%, which corresponds to G1 grade. The bigger lesion had a 28×24 mm macroscopic evaluation. However, histological analysis revealed an actual maximum diameter of 35 mm due to microscopic invasion. All margins were tumor-free. Duodenum and bile duct were tumor-free, and both tumors were limited to the pancreas. A total of 12 lymph nodes were retrieved in the specimen, all negative for malignancy. The patient was classified as a pT2N0M0, prognostic stage II, according to the Eighth Edition of the American Joint Committee on Cancer Staging Manual (AJCC)^3^.

## DISCUSSION AND REVIEW

### Indication for surgical treatment

**Figure 5:**
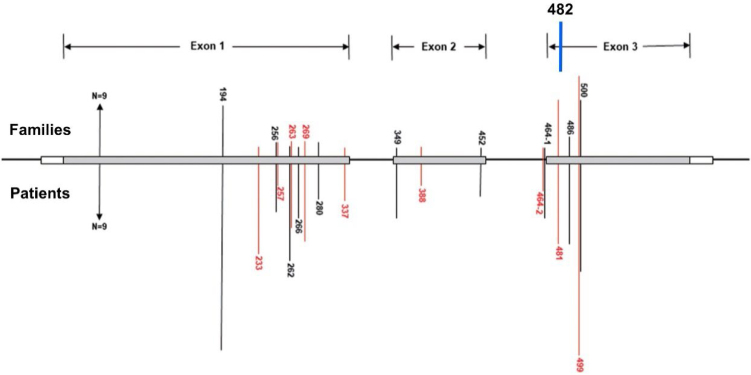
Distribution of the mutation sites in the von Hippel-Lindau gene for 258 patients from 80 unrelated Chinese families. The bars on the upper part represent mutational family numbers and those on the lower part represent mutational patient numbers. Our case mutation in the nucleotide 482 on the codon 161 (exon 3). Adapted from Hong et al.^14^.

Our patient has the mutation c.482G>A (p.Arg161Gln). Figure 5 illustrates our patient's mutation between the chromosomal abnormalities reported by Hong^13^. Undeniably, having a previously described genetic abnormality had facilitated his diagnosis. Nevertheless, physicians must remember that new pathological mutations on the VHL Suppressor Gene are still being discovered today.

Blansfield et al. suggested in 2007 that mutations in exon 3 of the VHL Suppressor Gene are related to a higher risk of malignant neuroendocrine pancreatic neoplasms, a higher risk of metastatic disease, and larger tumors^7^. Other studies endorsed these findings in the following years^8,12,16,22,23^. Nowadays, surgeons must consider three factors for indicating operative treatment in these patients^7,8,12,16,22,23^: (1) mutation in the exon 3, (2) tumors with 30 mm or larger, and (3) fast growth pattern (double size in 1 year or less).

Surgical treatment is formally indicated for patients with at least two of these three factors. Our patient had a confirmed mutation on exon 3, a 31-mm lesion on CT and MRI scans, and did not have serial abdominal imaging examinations for evaluating growth pattern. Although the tumor was only slightly above the upper limit for surgical indication, recent studies suggest that MRI is a highly precise tool for evaluating different densities and structural sizes^6^. Furthermore, the tumor was in the uncinate process, at the proximities of paramount vascular structures. We believe that even a slight tumor growth may have resulted in the need for an extended or more complex resection.

Recent studies reported that mutations on codon 167 of the exon 3 are at higher risk for metastatic disease^16,22^. Tirosh et al. studied 229 patients with VHL disease and reported that tumor size was closely related to the presence of metastasis^22^. The authors did not report a single case of metastatic disease in patients with tumors smaller than 12 mm. On the contrary, tumors larger than 30 mm had a high incidence of metastasis. Regarding patients with tumors between 12 and 30 mm, the presence of metastasis was related to missense mutations, which are mutations that result in different amino acid production. Our patient had mutations on codon 161 of the exon 3 and, despite having a 35 mm lesion on microscopic analysis, has not presented metastasis to date. The patient also has a missense mutation, inducing the production of glycine instead of arginine in the affected codon, which reinforced our choice of surgical resection instead of a watch and wait approach. He signed the informed consent form for this report.

### Imaging

Qiu et al.^20^. reported inaccuracy between CT scan and the actual tumor size in Eastern patients with adenocarcinoma, the most common pancreatic malignancy worldwide. Amico et al^2^. outlined the low accuracy of imaging diagnostic tools in the management of pancreatic cystic neoplasms. In a multicentric European study, Beleù et al. in 2019 reported comparable findings using CT and MRI scan in the preoperative evaluation of patients with pancreatic adenocarcinoma^4^.

Concerning pancreatic NETs imaging, in 2017, Paiella et al. reported a good correlation between the tumor size in preoperative CT and MRI scans and in the analysis of the surgical specimen of 292 patients. However, genetic syndromes and multiple NETs were exclusion criteria in their study, two conditions our patient presented^18^.

Multiple pancreatic NETs are a common condition in patients with VHL disease. Prasad et al. reported that 4 (36.4%) of the 11 patients with pancreatic NET in their VHL series presented more than one tumor^19^. Thus, surgeons must do a thorough preoperative evaluation focusing on the identification of all lesions. When there are multiple pancreatic NETs, all lesions that are 30 mm or larger have an indication for resection. Tumors smaller than 30 mm should be approached on a case-by-case basis. Surgeons must indicate the excision whenever it is possible to perform *en bloc* resection, but they also should avoid performing a total pancreatectomy on these patients. Enucleation of these smaller lesions is another option. However, preoperative imaging must clearly demonstrate at least a 3-mm margin free from the main pancreatic duct. In our patient, the *en bloc* resection of the smaller lesion identified on the PET-CT scan with DOTATOC-68Ga was feasible and straightforward. The DOTATOC-68Ga is a somatostatin analog marked with Gallium-68, which is an accurate radiotracer for evaluating and staging somatostatin-expressing tumors like NETs on the PET scan.

Angiotomography scan is an essential examination for evaluating tumor invasion and vascular anatomical variations. The most critical variant in pancreatic resections is a replaced right hepatic artery arising from the superior mesenteric artery and passing behind the head of the pancreas. This condition affects around 10% of the population and was not present in our patient.

### Technical aspects

Lopes et al. reviewed 695 pancreatic resections for NETs performed in eight institutions in the United States. This group concluded that adequate lymphadenectomy ameliorates staging and improves prognosis. They recommended at least seven lymph nodes for adequate nodal staging in distal pancreatectomies. They could not, however, recommend a minimum number of lymph nodes for adequate nodal staging in pancreatoduodenectomies for pancreatic NETs^17^.

Our core objectives in this operation were to resect both tumors with minimal bleeding, tumor-free margins, appropriate lymphadenectomy, and reconstruction with safe and functional anastomoses.

Tumors on the uncinate process sometimes require an artery-first approach, with opening of the Treitz ligament, dissection of the retroperitoneal space, and ligation of the branches of the superior mesenteric artery. In this case, we used a conventional approach, ligating the feeding branches of the superior mesenteric vein from the uncinate process and the head of the pancreas.

Patients with VHL disease are at higher risk for central nervous system hemangioblastomas, including in the spinal cord. Therefore, neuraxial anesthesia techniques must be used with caution in these patients^14^. Anesthesiologists prefer epidural anesthesia over spinal cord anesthesia when indicating neuraxial block in patients with VHL syndrome, for the danger of accidentally puncturing a hemangioblastoma is higher using a technique that enters the subarachnoid space. Berl et al. and Wang et al. reported no anesthetic complications when performing epidural anesthesia for cesarean sections in patients with VHL disease^5,24^.

Our patient did not have neurological symptoms or any signs of spinal lesions on imaging. The anesthesiology team indicated a lumbar epidural block, inserting a catheter for analgesia. They removed the catheter on the second postoperative day.

The COVID-19 pandemic in mid-2020 influenced the management of our patient. In a broad analysis, the pandemics overloaded the already vulnerable Brazilian public healthcare system, limiting the access to hospital facilities and technologies. Vaccines against COVID-19 were still unavailable then. In this individual case, the surgical team had to consider the possibility of a nosocomial COVID-19 infection, as it may have resulted in a worse surgical result and prognosis. We indicated surgery for the patient in mid-July 2020, but after considering the disease and the pandemics, we decided to postpone his operation to November 2020, after the burden on the healthcare system in our city had been reduced significantly^11^. A grade 1 NET is a less aggressive tumor, although still with metastatic potential. Obviously, if we were dealing with a more aggressive pancreatic neoplasm, such as an adenocarcinoma or a neuroendocrine carcinoma, postponing surgery for 4 months would not have been feasible.

To date, different medical specialties and societies worldwide still discuss the timing of surgery after a COVID-19 infection, for the patient or for someone in the household like in this case. CovidSurg, a multicenter international collaborative group for studying the impact of COVID-19 on surgical patients, published a consensus in June 2021 suggesting that a 7-week interval between infection and the operative treatment seems to be the safer option^9^. Further studies suggested that a 4-week interval would have been more adequate^10^.

The patient had a 21-day prolonged hospitalization. The main cause was the peripancreatic fluid collection, which we managed as a pancreatic fistula, even though it did not have a formal diagnosis, defined by an effluent amylase concentration of at least three times the serum concentration. This is a common complication after pancreaticoduodenectomy, particularly in patients with soft pancreatic parenchyma and nondilated main pancreatic duct. It is also noteworthy that the patient has had bilateral adrenalectomy previously and was dependent of glucocorticoid reposition for homeostasis, which may be a risk factor for postoperative infection and other complications.

Although the current literature assists in decision-making in most situations, each step of the treatment requires analysis and discussion between different medical specialties, including surgeons, clinicians, radiologists, and anesthesiologists. The COVID-19 pandemic was an important and unpredictable aspect when managing this patient, postponing but not worsening the quality of the assistance provided for him.

## CONCLUSION

The management of patients with VHL disease and pancreatic NETs is complex and must be centered on tertiary institutions with a large volume of pancreatic surgery.
